# Crystal structure of 5-chloro-2,4,6-trimethyl-3-(4-methyl­phenyl­sulfin­yl)-1-benzo­furan

**DOI:** 10.1107/S160053681402385X

**Published:** 2014-11-05

**Authors:** Hong Dae Choi, Uk Lee

**Affiliations:** a Department of Chemistry, Dongeui University, San 24 Kaya-dong, Busanjin-gu, Busan 614-714, Republic of Korea; b Department of Chemistry, Pukyong National University, 599-1 Daeyeon 3-dong, Nam-gu, Busan 608-737, Republic of Korea

**Keywords:** crystal structure, benzo­furan, 4-methyl­phen­yl, C—H⋯O and C—H⋯π hydrogen bonds, π–π and S⋯S inter­actions

## Abstract

In the title compound, C_18_H_17_ClO_2_S, the dihedral angle between the planes of the benzo­furan ring [r.m.s. deviation = 0.013 (1) Å] and the 4-methyl­phenyl ring is 87.37 (5)°. In the crystal, mol­ecules are linked by C—H⋯O hydrogen bonds and π–π inter­actions between the furan and benzene rings of neighbouring mol­ecules [centroid–centroid distance = 3.525 (2) Å]. In addition, an S⋯S [3.6584 (9) Å] contact is observed.

## Related literature   

For the pharmacological properties of benzo­furan compounds, see: Aslam *et al.* (2009 ▶); Galal *et al.* (2009 ▶); Howlett *et al.* (1999 ▶); Wahab Khan *et al.* (2005 ▶); Ono *et al.* (2002 ▶). For natural products with a benzo­furan ring, see: Akgul & Anil (2003 ▶); Soekamto *et al.* (2003 ▶). For the synthesis of the starting material 5-chloro-2,4,6-trimethyl-3-(4-methyl­phenyl­sulfan­yl)-1-benzo­furan, see: Choi *et al.* (1999 ▶). For a related structure, see: Choi *et al.* (2012 ▶).
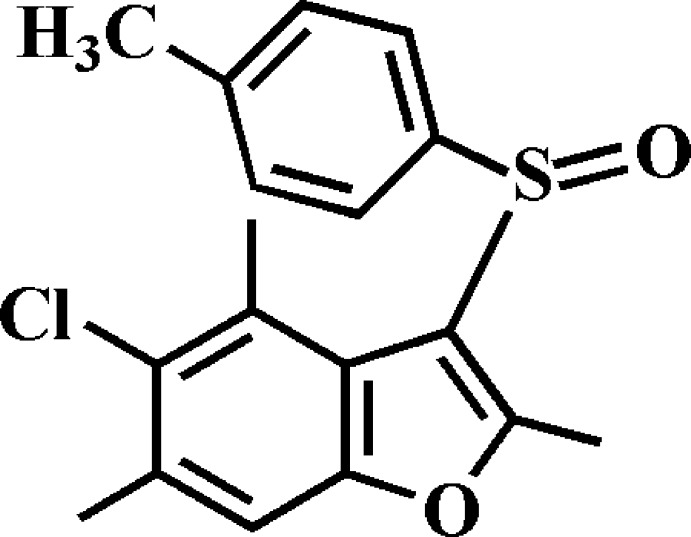


## Experimental   

### Crystal data  

C_18_H_17_ClO_2_S
*M* *_r_* = 332.83
Triclinic, 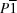

*a* = 8.7938 (2) Å
*b* = 9.1775 (3) Å
*c* = 10.7298 (3) Å
α = 86.571 (1)°
β = 69.157 (1)°
γ = 81.093 (2)°
*V* = 799.51 (4) Å^3^
*Z* = 2
Mo *K*α radiation
μ = 0.37 mm^−1^
*T* = 173 K
0.60 × 0.56 × 0.08 mm

### Data collection  

Bruker SMART APEXII CCD diffractometer
Absorption correction: multi-scan (*SADABS*; Bruker, 2009 ▶) *T*_min_ = 0.807, *T*_max_ = 0.971
14763 measured reflections
3988 independent reflections
3502 reflections with *I* > 2σ(*I*)
*R*_int_ = 0.048

### Refinement  

*R*[*F* ^2^ > 2σ(*F* ^2^)] = 0.043
*wR*(*F* ^2^) = 0.124
*S* = 1.05
3988 reflections
203 parameters
H-atom parameters constrained
Δρ_max_ = 0.83 e Å^−3^
Δρ_min_ = −0.41 e Å^−3^

### 

Data collection: *APEX2* (Bruker, 2009 ▶); cell refinement: *SAINT* (Bruker, 2009 ▶); data reduction: *SAINT*; program(s) used to solve structure: *SHELXS97* (Sheldrick, 2008 ▶); program(s) used to refine structure: *SHELXL97* (Sheldrick, 2008 ▶); molecular graphics: *ORTEP-3 for Windows* (Farrugia, 2012 ▶) and *DIAMOND* (Brandenburg, 1998 ▶); software used to prepare material for publication: *SHELXL97*.

## Supplementary Material

Crystal structure: contains datablock(s) I. DOI: 10.1107/S160053681402385X/nk2227sup1.cif

Structure factors: contains datablock(s) I. DOI: 10.1107/S160053681402385X/nk2227Isup2.hkl

Supporting information file. DOI: 10.1107/S160053681402385X/nk2227Isup3.cml

**Fig. 1.**
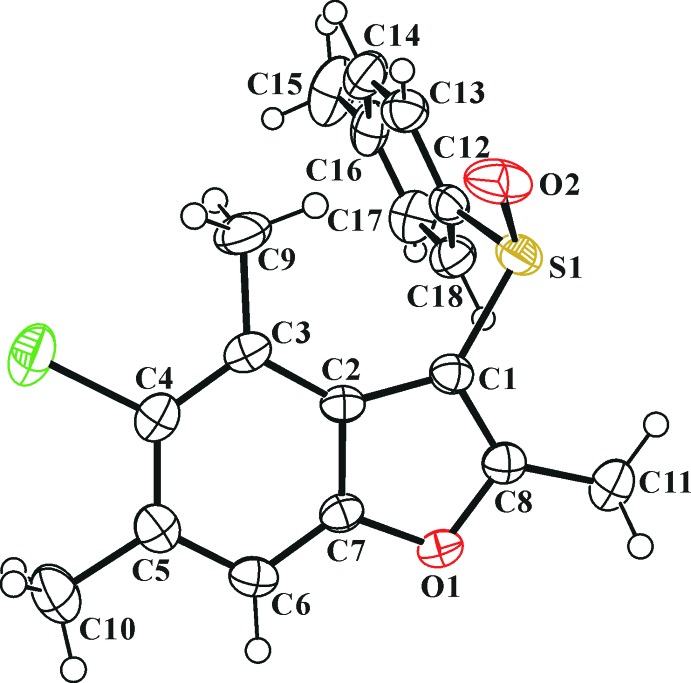
The molecular structure of the title compound with the atom numbering scheme. Displacement ellipsoids are drawn at the 50% probability level. H atoms are presented as small spheres of arbitrary radius.

**Fig. 2.**
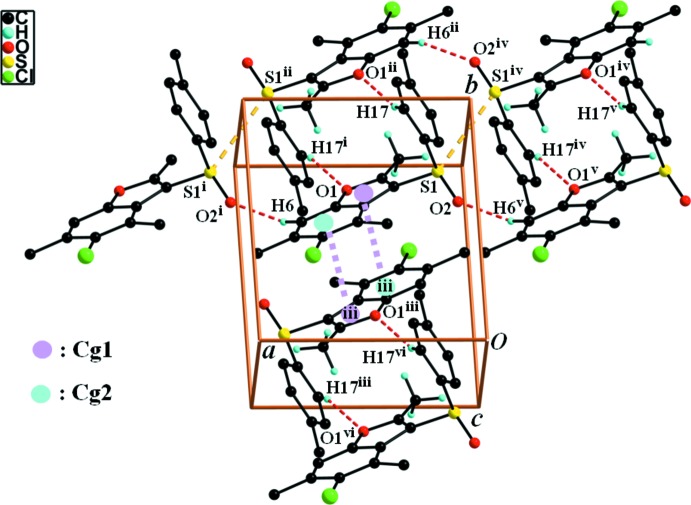
A view of the C—H···O, π–π and S···S interactions (dotted lines) in the crystal structure of the title compound. H atoms non-participating in hydrogen-bonding were omitted for clarity. [Symmetry codes: (i) x + 1, y, z; (ii) - x + 1, - y + 2, - z + 1; (iii) - x + 1, - y + 1, - z + 1; (iv) - x, - y + 2, - z + 1; (v) x - 1, y, z; (vi) x, y - 1, z.]

. DOI: 10.1107/S160053681402385X/nk2227fig1.tif
The mol­ecular structure of the title compound with the atom numbering scheme. Displacement ellipsoids are drawn at the 50% probability level. H atoms are presented as small spheres of arbitrary radius.

x y z x y z x y z x y z x y z x y z . DOI: 10.1107/S160053681402385X/nk2227fig2.tif
A view of the C—H⋯O, π–π and S⋯S inter­actions (dotted lines) in the crystal structure of the title compound. H atoms non-participating in hydrogen-bonding were omitted for clarity. [Symmetry codes: (i) *x* + 1, *y*, *z*; (ii) − *x* + 1, − *y* + 2, − *z* + 1; (iii) − *x* + 1, − *y* + 1, − *z* + 1; (iv) − *x*, − *y* + 2, − *z* + 1; (v) *x* − 1, *y*, *z*; (vi) *x*, *y* − 1, *z*.]

CCDC reference: 1031568

Additional supporting information:  crystallographic information; 3D view; checkCIF report

## Figures and Tables

**Table 1:** Hydrogen-bond geometry (, )

*D*H*A*	*D*H	H*A*	*D* *A*	*D*H*A*
C6H6O2^i^	0.95	2.30	3.180(2)	154
C17H17O1^ii^	0.95	2.56	3.434(2)	153

Symmetry codes: (i) 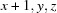; (ii) 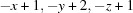.

## supplementary crystallographic information

### S1. Comment

Substituted benzofurans show interesting pharmacological properties such as antibacterial and antifungal, antitumor and antiviral, antimicrobial activities (Aslam *et al.* 2009, Galal *et al.*, 2009, Wahab Khan *et al.*, 2005), and potential inhibitor of β-amyloid aggregation (Howlett *et al.*, 1999, Ono *et al.*, 2002). These benzofuran compounds occur in a great number of natural products. (Akgul & Anil, 2003, Soekamto *et al.*, 2003). As a part of our ongoing project of 3-arylsulfinyl-5-chloro-2-methyl-1-benzofuran derivatives containing 4-bromophenylsulfinyl substituent in 3-position (Choi *et al.*, 2012), we report herein on the crystal structure of the title compound.

In the title molecule (Fig. 1), the benzofuran unit is essentially planar, with a mean deviation of 0.013 (1) Å from the least-squares plane defined by the nine constituent atoms. The 4-methylphenyl ring is essentially planar, with a mean deviation of 0.003 (1) Å from the least-squares plane defined by the six constituent atoms. The dihedral angle formed by the benzofuran ring and the 4-methylphenyl ring is 87.37 (5)°. In the crystal structure (Fig. 2), molecules are linked by C—H···O hydrogen bonds (Table 1) and π–π interactions between the furan and benzene rings of neighbouring molecules, with a Cg1···Cg2^iii^ distance of 3.525 (2) Å and an interplanar distance of 3.479 (2) Å resulting in a slippage of 0.568 (2) Å (Cg1 and Cg2 are the centroids of the C1/C2/C7/O1/C8 furan ring and the C2–C7 benzene ring, respectively). In the crystal (Fig. 2), an S1···S1^iv^ [3.6584 (9) Å] contact are observed.

### S2. Experimental

The starting material 5-chloro-2,4,6-trimethyl-3-(4-methylphenylsulfanyl)-1-benzofuran was prepared by literature method (Choi *et al.* 1999). 3-Chloroperoxybenzoic acid (77%, 224 mg, 1.0 mmol) was added in small portions to a stirred solution of 5-chloro-2,4,6-trimethyl-3-(4-methylphenylsulfanyl)-1-benzofuran (285 mg, 0.9 mmol) in dichloromethane (25 mL) at 273 K. After being stirred at room temperature for 8h, the mixture was washed with saturated sodium bicarbonate solution (2 X 10 mL) and the organic layer was separated, dried over magnesium sulfate, filtered and concentrated at reduced pressure. The residue was purified by column chromatography (hexane–ethyl acetate, 2:1 *v*/*v*) to afford the title compound as a colorless solid [yield 68% (226 mg); m.p. 466–467 K; *R*_f_ = 0.43 (hexane–ethyl acetate, 2:1 *v*/*v*)]. Single crystals suitable for X-ray diffraction were prepared by slow evaporation of a solution of the title compound (23 mg) in acetone (15 mL) at room temperature.

### S3. Refinement

All H atoms were positioned geometrically and refined using a riding model, with C—H = 0.95 Å for aryl and 0.98 Å for methyl H atoms, *U*_iso_ (H) = 1.2*U*_eq_ (C) for aryl and 1.5*U*_eq_ (C) for methyl H atoms.The positions of methyl hydrogens were optimized using the SHELXL-97's command AFIX 137 (Sheldrick, 2008).

### Crystal data

**Table:**

C_18_H_17_ClO_2_S	*Z* = 2
*M**_r_* = 332.83	*F*(000) = 348
Triclinic, *P*1	*D*_x_ = 1.383 Mg m^−^^3^
Hall symbol: -P 1	Melting point = 467–466 K
*a* = 8.7938 (2) Å	Mo *K*α radiation, λ = 0.71073 Å
*b* = 9.1775 (3) Å	Cell parameters from 6565 reflections
*c* = 10.7298 (3) Å	θ = 2.6–28.3°
α = 86.571 (1)°	µ = 0.37 mm^−^^1^
β = 69.157 (1)°	*T* = 173 K
γ = 81.093 (2)°	Block, colourless
*V* = 799.51 (4) Å^3^	0.60 × 0.56 × 0.08 mm

### Data collection

**Table:**

Bruker SMART APEXII CCD diffractometer	3988 independent reflections
Radiation source: rotating anode	3502 reflections with *I* > 2σ(*I*)
Graphite multilayer monochromator	*R*_int_ = 0.048
Detector resolution: 10.0 pixels mm^-1^	θ_max_ = 28.4°, θ_min_ = 2.0°
φ and ω scans	*h* = −11→11
Absorption correction: multi-scan (*SADABS*; Bruker, 2009)	*k* = −12→12
*T*_min_ = 0.807, *T*_max_ = 0.971	*l* = −11→14
14763 measured reflections	

### Refinement

**Table:**

Refinement on *F*^2^	Primary atom site location: structure-invariant direct methods
Least-squares matrix: full	Secondary atom site location: difference Fourier map
*R*[*F*^2^ > 2σ(*F*^2^)] = 0.043	Hydrogen site location: difference Fourier map
*wR*(*F*^2^) = 0.124	H-atom parameters constrained
*S* = 1.05	*w* = 1/[σ^2^(*F*_o_^2^) + (0.0641*P*)^2^ + 0.2785*P*] where *P* = (*F*_o_^2^ + 2*F*_c_^2^)/3
3988 reflections	(Δ/σ)_max_ < 0.001
203 parameters	Δρ_max_ = 0.83 e Å^−^^3^
0 restraints	Δρ_min_ = −0.41 e Å^−^^3^

### Special details

**Table:**

Experimental. ^1^H NMR (δ p.p.m., CDCl_3_, 400 Hz): 7.34 (d, J = 8.24 Hz, 2H), 7.22 (d, J = 7.84 Hz, 2H), 7.19 (s, 1H), 2.71 (s, 3H), 2.43 (s, 3H), 2.37 (s, 3H), 2.32 (s, 3H).
Geometry. All esds (except the esd in the dihedral angle between two l.s. planes) are estimated using the full covariance matrix. The cell esds are taken into account individually in the estimation of esds in distances, angles and torsion angles; correlations between esds in cell parameters are only used when they are defined by crystal symmetry. An approximate (isotropic) treatment of cell esds is used for estimating esds involving l.s. planes.
Refinement. Refinement of F^2^ against ALL reflections. The weighted R-factor wR and goodness of fit S are based on F^2^, conventional R-factors R are based on F, with F set to zero for negative F^2^. The threshold expression of F^2^ > 2sigma(F^2^) is used only for calculating R-factors(gt) etc. and is not relevant to the choice of reflections for refinement. R-factors based on F^2^ are statistically about twice as large as those based on F, and R- factors based on ALL data will be even larger.

### Fractional atomic coordinates and isotropic or equivalent isotropic displacement parameters (Å^2^)

**Table:**

	*x*	*y*	*z*	*U*_iso_*/*U*_eq_	
Cl1	0.73398 (7)	0.38071 (5)	0.09920 (5)	0.04987 (16)	
S1	0.14021 (5)	0.82594 (5)	0.46437 (4)	0.03532 (13)	
O1	0.54038 (13)	0.77389 (12)	0.54951 (11)	0.0305 (2)	
O2	0.06142 (17)	0.69774 (17)	0.45239 (15)	0.0502 (4)	
C1	0.33771 (18)	0.76626 (16)	0.46945 (16)	0.0281 (3)	
C2	0.47994 (18)	0.66515 (15)	0.39027 (15)	0.0256 (3)	
C3	0.5174 (2)	0.56896 (16)	0.28374 (16)	0.0298 (3)	
C4	0.6780 (2)	0.49579 (17)	0.23736 (16)	0.0321 (3)	
C5	0.7994 (2)	0.50928 (18)	0.29109 (17)	0.0330 (3)	
C6	0.75771 (19)	0.60170 (17)	0.39906 (16)	0.0315 (3)	
H6	0.8345	0.6131	0.4403	0.038*	
C7	0.60010 (19)	0.67636 (16)	0.44414 (15)	0.0265 (3)	
C8	0.38130 (19)	0.82609 (17)	0.56227 (16)	0.0296 (3)	
C9	0.3921 (3)	0.5445 (2)	0.2249 (2)	0.0471 (5)	
H9A	0.4114	0.5990	0.1406	0.071*	
H9B	0.2817	0.5794	0.2872	0.071*	
H9C	0.4015	0.4390	0.2084	0.071*	
C10	0.9703 (2)	0.4263 (2)	0.2351 (2)	0.0486 (5)	
H10A	1.0322	0.4416	0.2921	0.073*	
H10B	1.0256	0.4623	0.1449	0.073*	
H10C	0.9645	0.3209	0.2320	0.073*	
C11	0.2955 (2)	0.9357 (2)	0.66964 (18)	0.0405 (4)	
H11A	0.3379	1.0297	0.6427	0.061*	
H11B	0.3145	0.9000	0.7515	0.061*	
H11C	0.1773	0.9499	0.6857	0.061*	
C12	0.19683 (19)	0.91671 (17)	0.30487 (16)	0.0303 (3)	
C13	0.1421 (2)	0.87745 (19)	0.20800 (19)	0.0374 (4)	
H13	0.0840	0.7954	0.2211	0.045*	
C14	0.1729 (2)	0.9595 (2)	0.09110 (19)	0.0426 (4)	
H14	0.1359	0.9324	0.0238	0.051*	
C15	0.2563 (3)	1.07992 (19)	0.07025 (18)	0.0433 (4)	
C16	0.3090 (3)	1.11627 (19)	0.1702 (2)	0.0448 (4)	
H16	0.3671	1.1982	0.1576	0.054*	
C17	0.2795 (2)	1.03701 (19)	0.28696 (19)	0.0383 (4)	
H17	0.3155	1.0645	0.3547	0.046*	
C18	0.2873 (4)	1.1712 (2)	−0.0548 (2)	0.0668 (7)	
H18A	0.4004	1.1425	−0.1153	0.100*	
H18B	0.2711	1.2758	−0.0323	0.100*	
H18C	0.2105	1.1550	−0.0983	0.100*	

### Atomic displacement parameters (Å^2^)

**Table:**

	*U*^11^	*U*^22^	*U*^33^	*U*^12^	*U*^13^	*U*^23^
Cl1	0.0693 (3)	0.0399 (2)	0.0368 (2)	0.0026 (2)	−0.0170 (2)	−0.01002 (18)
S1	0.0252 (2)	0.0470 (3)	0.0337 (2)	−0.00534 (16)	−0.01096 (16)	0.00375 (17)
O1	0.0335 (6)	0.0326 (5)	0.0308 (6)	−0.0075 (4)	−0.0165 (5)	−0.0015 (4)
O2	0.0404 (7)	0.0647 (9)	0.0563 (9)	−0.0267 (6)	−0.0252 (7)	0.0181 (7)
C1	0.0271 (7)	0.0302 (7)	0.0285 (7)	−0.0066 (6)	−0.0109 (6)	0.0033 (6)
C2	0.0271 (7)	0.0255 (6)	0.0278 (7)	−0.0082 (5)	−0.0129 (6)	0.0045 (5)
C3	0.0373 (8)	0.0276 (7)	0.0295 (8)	−0.0077 (6)	−0.0168 (7)	0.0012 (6)
C4	0.0420 (9)	0.0266 (7)	0.0274 (7)	−0.0058 (6)	−0.0116 (7)	0.0016 (6)
C5	0.0314 (8)	0.0323 (8)	0.0312 (8)	−0.0045 (6)	−0.0072 (6)	0.0067 (6)
C6	0.0292 (7)	0.0346 (8)	0.0342 (8)	−0.0089 (6)	−0.0146 (6)	0.0059 (6)
C7	0.0298 (7)	0.0264 (7)	0.0265 (7)	−0.0085 (5)	−0.0123 (6)	0.0024 (5)
C8	0.0309 (7)	0.0297 (7)	0.0293 (8)	−0.0062 (6)	−0.0117 (6)	0.0031 (6)
C9	0.0541 (11)	0.0450 (10)	0.0563 (12)	−0.0038 (8)	−0.0359 (10)	−0.0133 (9)
C10	0.0346 (9)	0.0530 (11)	0.0465 (11)	0.0025 (8)	−0.0043 (8)	0.0032 (9)
C11	0.0484 (10)	0.0385 (9)	0.0327 (9)	−0.0041 (7)	−0.0122 (8)	−0.0044 (7)
C12	0.0262 (7)	0.0323 (7)	0.0310 (8)	0.0008 (6)	−0.0103 (6)	0.0003 (6)
C13	0.0385 (9)	0.0350 (8)	0.0419 (9)	−0.0027 (7)	−0.0190 (8)	−0.0017 (7)
C14	0.0548 (11)	0.0390 (9)	0.0354 (9)	0.0051 (8)	−0.0216 (8)	−0.0067 (7)
C15	0.0565 (11)	0.0307 (8)	0.0335 (9)	0.0074 (7)	−0.0097 (8)	−0.0024 (7)
C16	0.0544 (11)	0.0300 (8)	0.0477 (11)	−0.0071 (8)	−0.0149 (9)	0.0007 (7)
C17	0.0429 (9)	0.0356 (8)	0.0399 (9)	−0.0045 (7)	−0.0190 (8)	−0.0032 (7)
C18	0.105 (2)	0.0425 (11)	0.0395 (11)	0.0014 (12)	−0.0150 (12)	0.0042 (9)

### Geometric parameters (Å, º)

**Table:**

Cl1—C4	1.7500 (16)	C9—H9C	0.9800
S1—O2	1.4882 (14)	C10—H10A	0.9800
S1—C1	1.7574 (15)	C10—H10B	0.9800
S1—C12	1.7950 (17)	C10—H10C	0.9800
S1—S1^i^	3.6584 (9)	C11—H11A	0.9800
O1—C8	1.3672 (19)	C11—H11B	0.9800
O1—C7	1.3813 (18)	C11—H11C	0.9800
C1—C8	1.357 (2)	C12—C13	1.377 (2)
C1—C2	1.458 (2)	C12—C17	1.381 (2)
C2—C7	1.392 (2)	C13—C14	1.386 (3)
C2—C3	1.399 (2)	C13—H13	0.9500
C3—C4	1.393 (2)	C14—C15	1.384 (3)
C3—C9	1.501 (2)	C14—H14	0.9500
C4—C5	1.405 (2)	C15—C16	1.386 (3)
C5—C6	1.384 (2)	C15—C18	1.501 (3)
C5—C10	1.502 (2)	C16—C17	1.372 (3)
C6—C7	1.376 (2)	C16—H16	0.9500
C6—H6	0.9500	C17—H17	0.9500
C8—C11	1.481 (2)	C18—H18A	0.9800
C9—H9A	0.9800	C18—H18B	0.9800
C9—H9B	0.9800	C18—H18C	0.9800
			
O2—S1—C1	110.50 (8)	C5—C10—H10A	109.5
O2—S1—C12	106.80 (8)	C5—C10—H10B	109.5
C1—S1—C12	98.98 (7)	H10A—C10—H10B	109.5
O2—S1—S1^i^	113.79 (6)	C5—C10—H10C	109.5
C1—S1—S1^i^	134.58 (5)	H10A—C10—H10C	109.5
C12—S1—S1^i^	77.68 (5)	H10B—C10—H10C	109.5
C8—O1—C7	106.51 (11)	C8—C11—H11A	109.5
C8—C1—C2	107.06 (13)	C8—C11—H11B	109.5
C8—C1—S1	118.18 (12)	H11A—C11—H11B	109.5
C2—C1—S1	134.71 (12)	C8—C11—H11C	109.5
C7—C2—C3	118.93 (14)	H11A—C11—H11C	109.5
C7—C2—C1	104.37 (13)	H11B—C11—H11C	109.5
C3—C2—C1	136.70 (14)	C13—C12—C17	120.71 (16)
C4—C3—C2	115.52 (14)	C13—C12—S1	120.19 (13)
C4—C3—C9	122.27 (15)	C17—C12—S1	118.68 (13)
C2—C3—C9	122.20 (15)	C12—C13—C14	119.00 (17)
C3—C4—C5	125.18 (15)	C12—C13—H13	120.5
C3—C4—Cl1	117.32 (12)	C14—C13—H13	120.5
C5—C4—Cl1	117.49 (13)	C15—C14—C13	121.47 (17)
C6—C5—C4	118.13 (15)	C15—C14—H14	119.3
C6—C5—C10	119.95 (16)	C13—C14—H14	119.3
C4—C5—C10	121.92 (17)	C14—C15—C16	117.83 (17)
C7—C6—C5	117.09 (14)	C14—C15—C18	121.9 (2)
C7—C6—H6	121.5	C16—C15—C18	120.3 (2)
C5—C6—H6	121.5	C17—C16—C15	121.75 (18)
C6—C7—O1	124.07 (13)	C17—C16—H16	119.1
C6—C7—C2	125.10 (14)	C15—C16—H16	119.1
O1—C7—C2	110.83 (13)	C16—C17—C12	119.23 (16)
C1—C8—O1	111.21 (13)	C16—C17—H17	120.4
C1—C8—C11	133.88 (15)	C12—C17—H17	120.4
O1—C8—C11	114.89 (14)	C15—C18—H18A	109.5
C3—C9—H9A	109.5	C15—C18—H18B	109.5
C3—C9—H9B	109.5	H18A—C18—H18B	109.5
H9A—C9—H9B	109.5	C15—C18—H18C	109.5
C3—C9—H9C	109.5	H18A—C18—H18C	109.5
H9A—C9—H9C	109.5	H18B—C18—H18C	109.5
H9B—C9—H9C	109.5		
			
O2—S1—C1—C8	−134.63 (14)	C8—O1—C7—C2	−0.45 (16)
C12—S1—C1—C8	113.57 (14)	C3—C2—C7—C6	1.5 (2)
S1^i^—S1—C1—C8	32.06 (17)	C1—C2—C7—C6	−178.85 (15)
O2—S1—C1—C2	48.36 (18)	C3—C2—C7—O1	−178.86 (13)
C12—S1—C1—C2	−63.44 (17)	C1—C2—C7—O1	0.82 (16)
S1^i^—S1—C1—C2	−144.96 (13)	C2—C1—C8—O1	0.66 (18)
C8—C1—C2—C7	−0.88 (17)	S1—C1—C8—O1	−177.12 (10)
S1—C1—C2—C7	176.36 (13)	C2—C1—C8—C11	179.10 (17)
C8—C1—C2—C3	178.70 (17)	S1—C1—C8—C11	1.3 (3)
S1—C1—C2—C3	−4.1 (3)	C7—O1—C8—C1	−0.16 (17)
C7—C2—C3—C4	−2.4 (2)	C7—O1—C8—C11	−178.91 (13)
C1—C2—C3—C4	178.08 (16)	O2—S1—C12—C13	10.13 (16)
C7—C2—C3—C9	176.67 (16)	C1—S1—C12—C13	124.85 (14)
C1—C2—C3—C9	−2.9 (3)	S1^i^—S1—C12—C13	−101.30 (13)
C2—C3—C4—C5	1.6 (2)	O2—S1—C12—C17	−177.26 (13)
C9—C3—C4—C5	−177.46 (17)	C1—S1—C12—C17	−62.55 (14)
C2—C3—C4—Cl1	−177.10 (11)	S1^i^—S1—C12—C17	71.31 (13)
C9—C3—C4—Cl1	3.8 (2)	C17—C12—C13—C14	0.8 (2)
C3—C4—C5—C6	0.3 (3)	S1—C12—C13—C14	173.25 (13)
Cl1—C4—C5—C6	179.01 (12)	C12—C13—C14—C15	−0.4 (3)
C3—C4—C5—C10	179.99 (16)	C13—C14—C15—C16	0.2 (3)
Cl1—C4—C5—C10	−1.3 (2)	C13—C14—C15—C18	−178.73 (18)
C4—C5—C6—C7	−1.3 (2)	C14—C15—C16—C17	−0.4 (3)
C10—C5—C6—C7	178.96 (15)	C18—C15—C16—C17	178.56 (19)
C5—C6—C7—O1	−179.13 (14)	C15—C16—C17—C12	0.8 (3)
C5—C6—C7—C2	0.5 (2)	C13—C12—C17—C16	−1.0 (3)
C8—O1—C7—C6	179.22 (15)	S1—C12—C17—C16	−173.55 (13)

Symmetry code: (i) −*x*, −*y*+2, −*z*+1.

### Hydrogen-bond geometry (Å, º)

**Table:**

*D*—H···*A*	*D*—H	H···*A*	*D*···*A*	*D*—H···*A*
C6—H6···O2^ii^	0.95	2.30	3.180 (2)	154
C17—H17···O1^iii^	0.95	2.56	3.434 (2)	153

Symmetry codes: (ii) *x*+1, *y*, *z*; (iii) −*x*+1, −*y*+2, −*z*+1.
