# Supplementary figures and images for: Crystal structure of 5-chloro-2,4,6-trimethyl-3-(4-methyl­phenyl­sulfin­yl)-1-benzo­furan
Source: Acta Crystallogr Sect E Struct Rep Online. 2014 Nov 5;70(Pt 12):o1233–4. doi: 10.1107/S160053681402385X (PMC4257403; doi:10.1107/S160053681402385X)

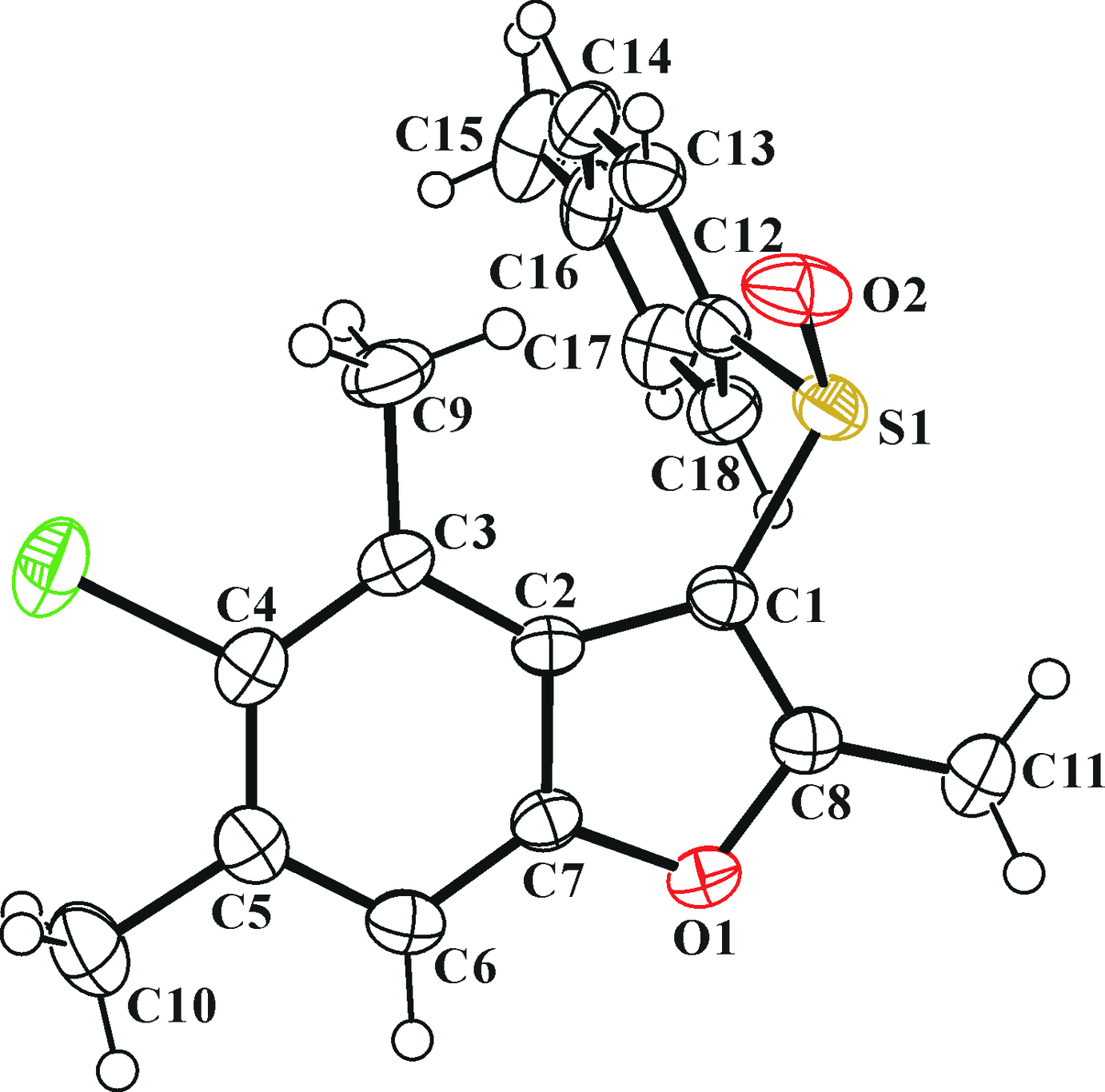

Supplement: Supplementary file 4 [file e-70-o1233-fig1.tif]

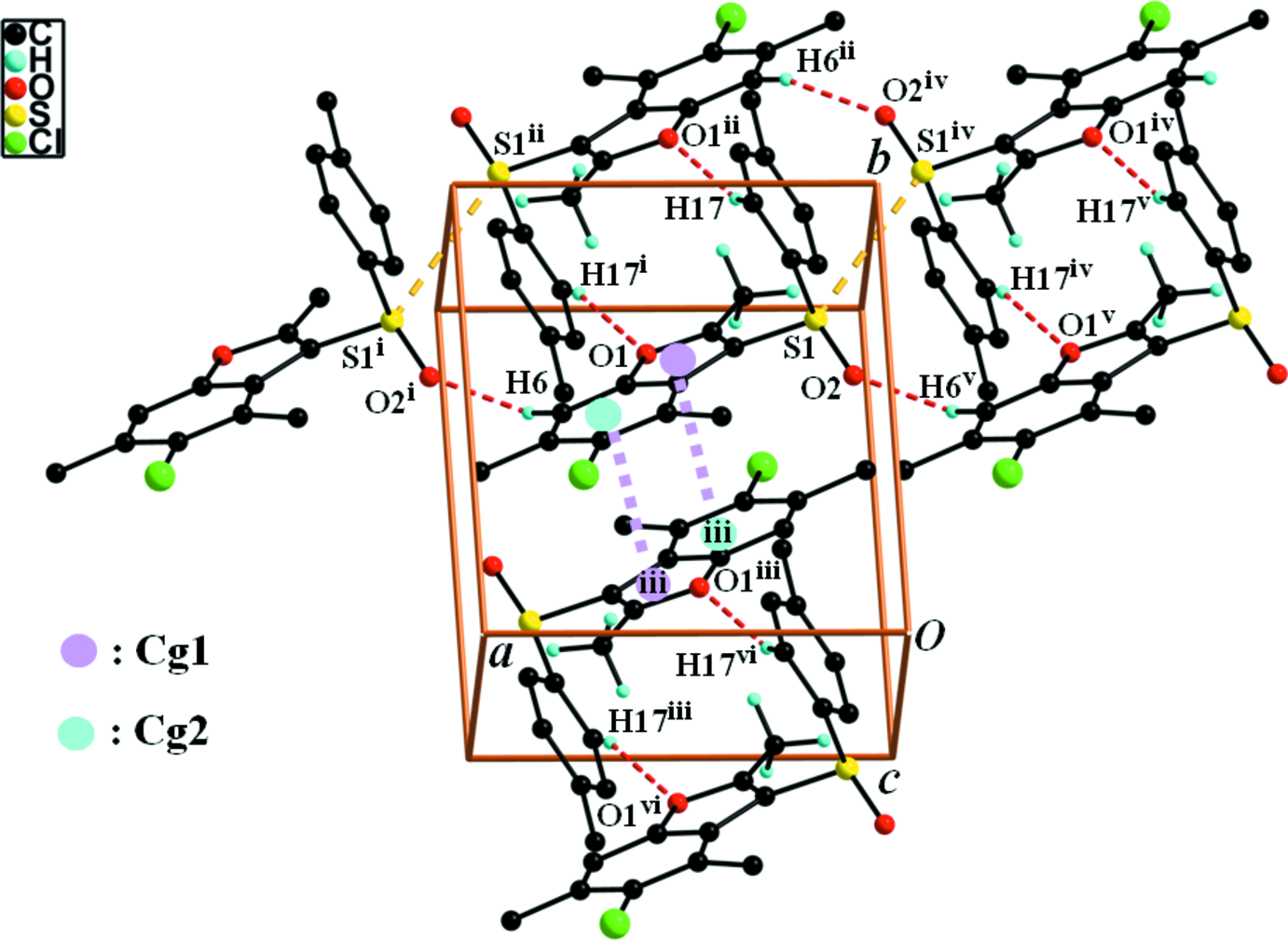

Supplement: Supplementary file 5 [file e-70-o1233-fig2.tif]
